# Cerebrovascular Malformations Associated With Hereditary Hemorrhagic Telangiectasia and HHT‐Like Syndromes: A Comparative Overview

**DOI:** 10.1111/ene.70523

**Published:** 2026-02-18

**Authors:** Matteo Palermo, Carmelo Lucio Sturiale

**Affiliations:** ^1^ Department of Neurosurgery Fondazione Policlinico Universitario A. Gemelli IRCCS, Università Cattolica del Sacro Cuore Rome Italy

**Keywords:** AVM, cerebrovascular malformations, hereditary hemorrhagic telangiectasia, HHT‐like, vascular phenotypes

## Abstract

**Introduction:**

Hereditary hemorrhagic telangiectasia (HHT) is an autosomal dominant vascular disorder marked by mucocutaneous telangiectasias, recurrent epistaxis, and visceral arteriovenous malformations (AVMs). Neurologic risks include brain AVMs and hemorrhagic stroke. Several rare genetic and sporadic syndromes (“HHT‐like” syndromes) share overlapping vascular features, complicating diagnosis. Differentiating these conditions is essential for accurate neurovascular risk assessment.

**Methods:**

A comprehensive literature review (PubMed, Scopus, Embase, Google Scholar; 1990–2025) targeted cerebrovascular manifestations of HHT and related syndromes. Key entities included Wyburn–Mason syndrome, Cobb syndrome, Klippel–Trénaunay syndrome (KTS), neurofibromatosis type 1 (NF1), PHACE(S) syndrome, capillary malformation–AVM (CM‐AVM), Parkes Weber syndrome (PWS), juvenile polyposis/HHT overlap (JP‐HHT), HHT type 5 (BMP9/GDF2), PTEN hamartoma tumor syndrome (PHTS), and blue rubber bleb nevus syndrome (BRBNS). Data on gene variants, lesion types, neuroimaging, stroke risk, and neurologic outcomes were synthesized.

**Results:**

High‐flow cerebrovascular malformations similar to HHT are prominent in Wyburn–Mason syndrome, CM‐AVM, and PWS, conferring a substantial hemorrhagic stroke risk. NF1 and PHACE(S) primarily feature occlusive arteriopathies linked to ischemic events. KTS, BRBNS, and PHTS predominantly show low‐ or mixed‐flow anomalies with lower CNS hemorrhagic risk but increased thrombotic complications. JP‐HHT carries added gastrointestinal cancer risk via SMAD4 variants, while HHT type 5 often presents incompletely. Genetic testing and tailored neuroimaging are critical for differentiation.

**Conclusions:**

Although many syndromes mimic HHT, few combine mucosal telangiectasias, high‐flow AVMs, and recurrent hemorrhage. Integrating clinical, imaging, and genetic data enables precise diagnosis, risk stratification, and personalized management.

## Graphical abstract

Hereditary hemorrhagic telangiectasia (HHT) and several HHT‐like syndromes, including Wyburn–Mason, Cobb, Klippel–Trénaunay, Parkes Weber, neurofibromatosis type 1, PHACE(S), capillary malformation–AVM (CM‐AVM), Juvenile polyposis/HHT overlap, HHT type 5, PTEN hamartoma tumor syndrome, and blue rubber bleb nevus syndrome, share overlapping cerebrovascular features that complicate diagnosis. This review compares their genetic basis, vascular flow types, and neurologic risks, highlighting distinctions between high‐flow AVMs, occlusive arteriopathies, and low‐ or mixed‐flow malformations.

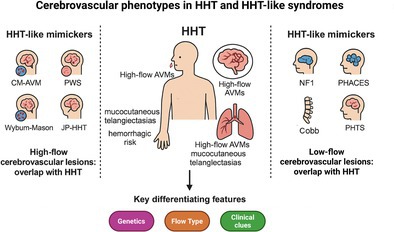

**Abbreviations**
AVF: arteriovenous fistula
AVM: arteriovenous malformation
BRBNS: blue rubber bleb nevus syndrome
CM‐AVM: capillary malformation–arteriovenous malformation syndrome
CNS: central nervous system
DVA: developmental venous anomaly
HHT: hereditary hemorrhagic telangiectasia
JP‐HHT: juvenile polyposis–hereditary hemorrhagic telangiectasia overlap syndrome
KTS: Klippel–Trénaunay syndrome
MRA: magnetic resonance angiography
MRI: magnetic resonance imaging
NF1: neurofibromatosis type 1
PHACES: posterior fossa anomalies, hemangioma, arterial anomalies, cardiac anomalies, eye anomalies, and sternal defects
PHTS: PTEN hamartoma tumor syndrome
PROS: PIK3CA‐related overgrowth spectrum
PWS: Parkes Weber syndrome
WMS: Wyburn–Mason syndrome

## 1 Introduction

Hereditary hemorrhagic telangiectasia (HHT), also known as Osler‐Weber‐Rendu disease, is an autosomal dominant vascular disorder characterized by recurrent hemorrhages and multiple telangiectases on skin and mucous membranes [1]. Visceral arteriovenous malformations (AVMs) frequently occur in the lungs, liver, brain, and gastrointestinal tract. *ENG* (endoglin, OMIN: 187300) and *ACVRL1* (ALK1, OMIM: 600376) gene mutations are associated with HHT types 1 and 2, while other rarer gene variants such as *SMAD4 (OMIM: 175050)* and GDF2/BMP9 (OMIM: 615506) may cause HHT variants or overlap syndromes. Clinical diagnosis of HHT is based on the Curaçao criteria, which include: spontaneous, recurrent epistaxis; multiple mucocutaneous telangiectases at characteristic lips, oral cavity, fingers and nose; visceral AVMs (pulmonary, hepatic, cerebral, or gastrointestinal); and a first‐degree relative with HHT [2]. Definite HHT requires 3 of 4 criteria; possible HHT requires 2. However, despite these diagnostic criteria, HHT is still highly under‐diagnosed, and many patients come to clinical attention only after complications. For this reason, ENG and ACVRL1 have been added in the ACMG list of actionable incidental findings, so that carriers identified through exome or genome sequencing can receive early clinical and neuroradiological surveillance [3].

HHT is characterized by a distinctive cerebrovascular profile, mainly characterized by high‐flow vascular malformations such as pial AVMs in up to 20% of patients, occasional dural arteriovenous fistulas (dAVFs), and rare spinal AVMs. These lesions can lead to a risk of hemorrhagic stroke and epilepsy. Non‐flow related aneurysms and low flow vascular malformations such as cavernous angiomas and, more rarely, developmental venous anomalies (DVAs) may also be found in patients with HHT, though this can be more a casual than a causal association [2, 4]. The screening with neuroimaging, especially MRI/MRA, is recommended for all affected individuals and at‐risk relatives.

Several other genetic conditions can be associated with cerebrovascular phenotypes mimicking HHT syndrome. These conditions share with HHT patients the hemorrhagic risk not only at the level of the central nervous system (CNS) but also at mucocutaneous and visceral organs, and frequently need to be placed in differential diagnosis [5].

In this review, we discuss a group of clinically similar disorders collectively known as “HHT‐like” syndromes. We place particular emphasis on both their shared and distinguishing neurovascular manifestations, aiming to help clinicians and researchers recognize phenotypic patterns and avoid potential diagnostic pitfalls [5].

## 2 Methods

A literature review was conducted to gather and synthesize the most relevant evidence on the cerebrovascular manifestations of HHT and phenotypically related disorders collectively referred to as “HHT‐like” syndromes. The objective was to describe and compare the prevalence, types, and clinical significance of AVMs, dAVFs, DVAs, cerebral aneurysms, cavernomas, stroke, and epilepsy across these conditions. Searches were performed in multiple databases such as PubMed, Scopus, Embase, and Google Scholar, covering publications from January 1990 to March 2025. Keyword combinations included “hereditary hemorrhagic telangiectasia OR HHT,” “Osler‐Weber‐Rendu,” “ENG,” “ACVRL1,” “SMAD4,” “GDF2,” “BMP9,” “capillary malformation arteriovenous malformation syndrome OR CM‐AVM,” “RASA1,” “EPHB4,” “Parkes Weber syndrome,” “juvenile polyposis,” “PIK3CA‐related overgrowth,” “CLOVES syndrome,” “blue rubber bleb nevus syndrome,” “BRBNS,” “TEK,” “PTEN hamartoma tumor syndrome,” “Cowden syndrome,” “Klippel–Trénaunay syndrome,” “Wyburn–Mason syndrome,” “Cobb syndrome,” “Neurofibromatosis type 1,” “NF1,” and “PHACES syndrome.”

The aim of this search was to create a comprehensive comparative overview of these genetic syndromes sharing similar cerebrovascular phenotypes.

### 2.1 Selection Criteria

We included original research articles, reviews, case series, and relevant case reports that described cerebrovascular disorders in any of the aforementioned syndromes. Only articles written in English were considered, and studies focusing exclusively on non‐neurological vascular manifestations without reference to CNS involvement were excluded. Animal studies and conference abstracts were also excluded. Retrieved articles were initially screened based on title and abstract relevance, followed by full‐text review of the selected papers. Priority was given to studies reporting on prevalence, imaging findings, or pathophysiologic mechanisms of cerebrovascular anomalies.

### 2.2 Data Extraction

**FIGURE 1:**
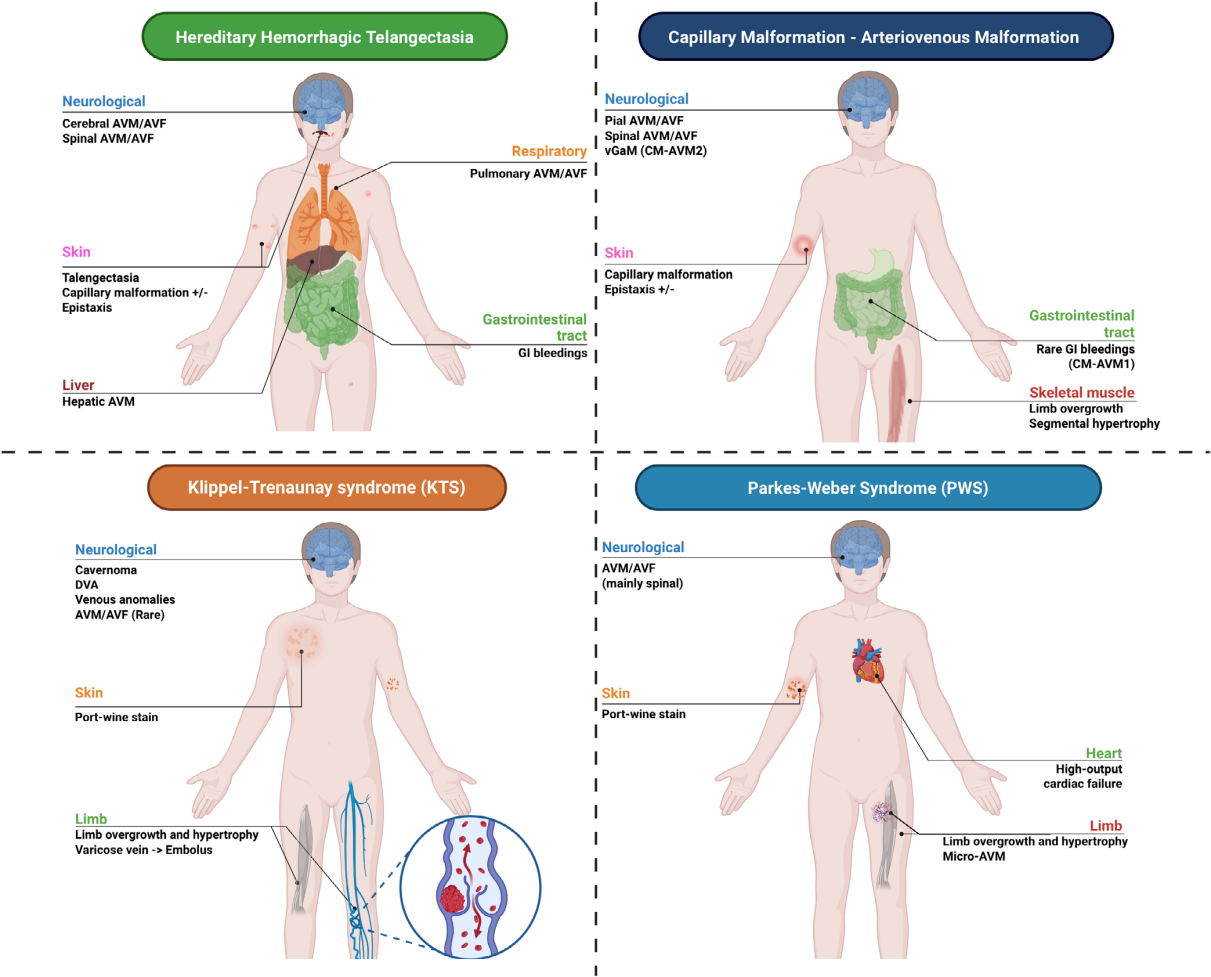
Schematic representation of systemic and neurological manifestations in Juvenile polyposis/hereditary hemorrhagic telangiectasia (HHT) overlap, HHT type 5 (GDF2/BMP9), PTEN hamartoma tumor syndrome, and blue rubber bleb nevus syndrome.

**FIGURE 2:**
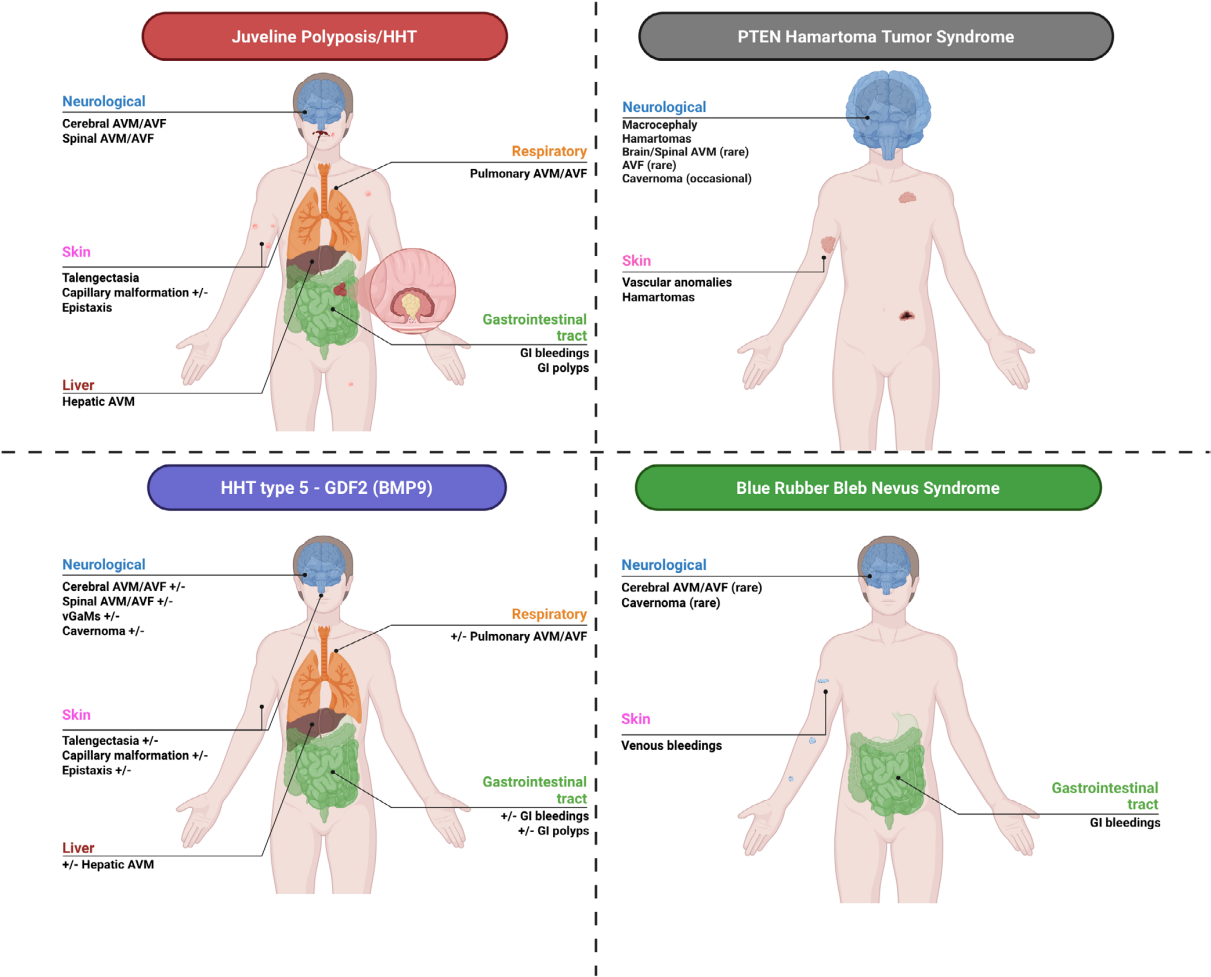
Schematic overview of multisystem vascular malformations in hereditary hemorrhagic telangiectasia (HHT), capillary malformation–arteriovenous malformation syndrome (CM‐AVM), Klippel–Trénaunay syndrome (KTS), and Parkes–Weber syndrome (PWS).

**FIGURE 3:**
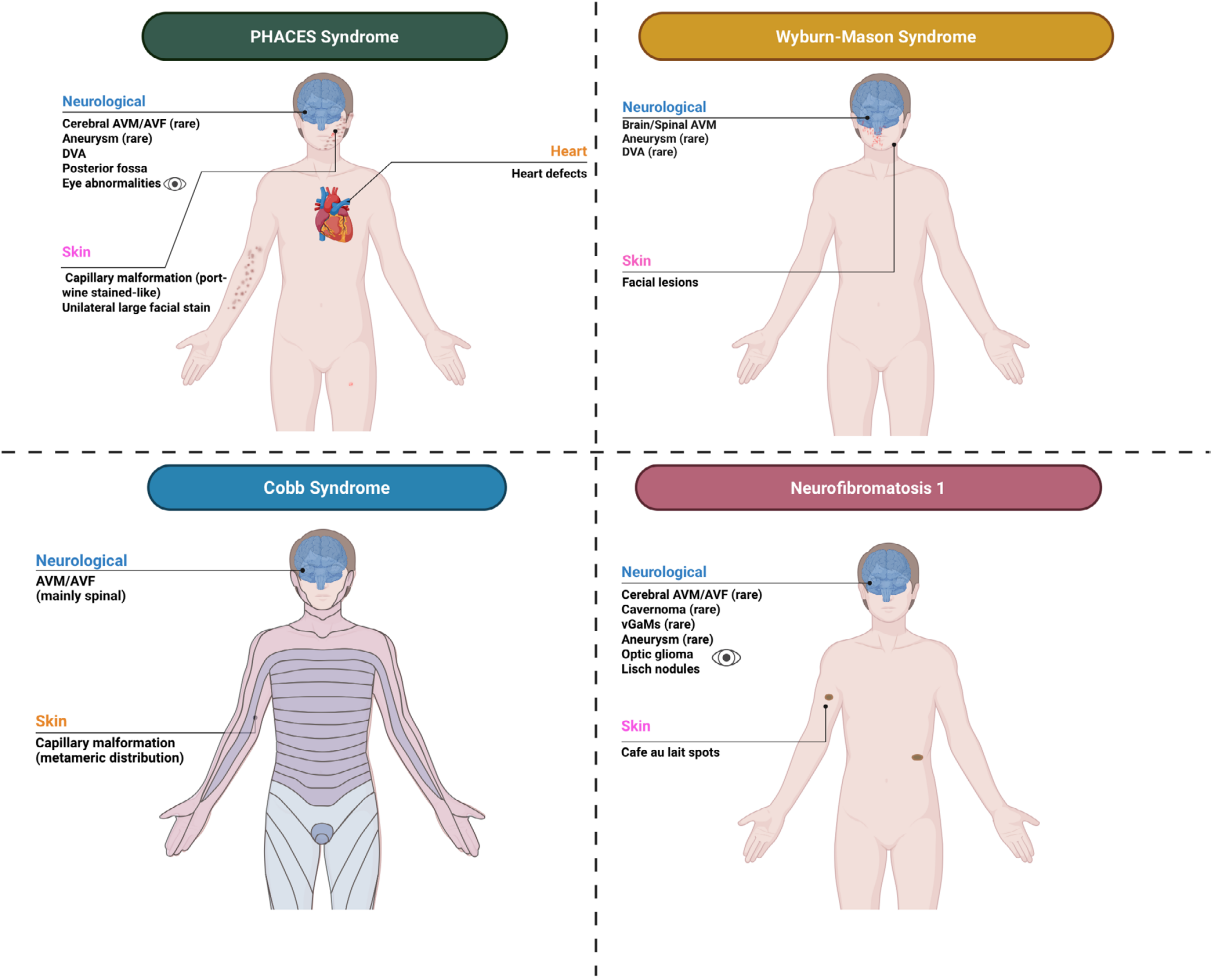
Schematic representation of vascular and neurological manifestations in PHACES syndrome, Wyburn–Mason syndrome, Cobb syndrome, and neurofibromatosis type 1.

Data were extracted from the selected publications and systematically organized into structured Tables 1 and 2, which summarize the clinical, genetic, and cerebrovascular characteristics of HHT and HHT‐like syndromes. For each syndrome, the following information was recorded: gene variant and inheritance pattern; overlapping and distinguishing clinical features; prevalence and anatomical distribution of cerebrovascular malformations; type of cerebrovascular flow; occurrence of ischemic or hemorrhagic stroke; and associated neurologic manifestations. These variables were compiled into comparative tables and used to generate narrative summaries for each syndrome. Additionally, Figures 1, 2, 3 provide graphical representations of the various HHT‐like syndromes to facilitate visual comparison and support data synthesis.

**TABLE 1:** Clinical and genetic features differentiating HHT from related vascular syndromes.

Syndrome/condition	Gene mutation (inheritance)	OMIM	Overlapping features with HHT	Key distinguishing features
Hereditary hemorrhagic telangiectasia (HHT) (Osler‐Weber‐Rendu)	ENG (HHT1), ACVRL1 (HHT2), etc. (AD)	187300/600376	Baseline—telangiectases on lips, oral/nasal mucosa, fingertips; recurrent epistaxis; AVMs in lung, liver, brain; family history often positive.	Curaçao criteria (3 of 4). Genetic cause in TGF‐β pathway. Distinguished by combination of mucocutaneous telangiectasia + visceral AVMs + dominant inheritance. (Used as reference standard in this table.)
Capillary malformation–AVM syndrome (CM‐AVM1)—RASA1 mutation	RASA1 (AD)	608354	Cutaneous vascular lesions (capillary malformations) and brain AVMs—overlaps with HHT's skin telangiectasia and cerebral AVMs. Epistaxis can occur (some RASA1 patients have nosebleeds).	Capillary malformations (CMs)—typically larger, flat pink‐red patches on skin present from birth (port‐wine‐like) on face/trunk/limbs. Telangiectases may be more numerous and distributed on trunk/limbs, not predominantly lips/oral as in HHT. Limb overgrowth or Parkes Weber (due to underlying AV fistulas) may occur, which HHT does not cause. Pulmonary AVMs are uncommon (vs 50% in HHT1). Genetic testing distinguishes (RASA1 vs. ENG/ACVRL1).
Capillary malformation–AVM syndrome (CM‐AVM2)—EPHB4 mutation	EPHB4 (AD)	618196	Multifocal telangiectatic CMs, epistaxis, and AVMs—can fulfill HHT clinical criteria (hence “HHT‐like”). Some have hepatic AVM/shunting similar to HHT.	Vein of Galen malformation in infancy is a tip‐off for EPHB4 (not seen in HHT). Capillary lesions from birth (as in RASA1). Possibly fewer parenchymal brain AVMs overall, but notably more frequent nasal telangiectases and nosebleeds than RASA1—yet still, context of capillary stains on body distinguishes from HHT. Genetic test for EPHB4. Basically, look for port‐wine‐like lesions + epistaxis + negative ENG/ACVRL1.
Juvenile polyposis/HHT overlap (JP‐HHT)—SMAD4 mutation	SMAD4 (AD)	610655/175050	All classic HHT features (epistaxis, telangiectasia, AVMs) are present—vascular phenotype same as HHT.	Multiple juvenile polyps in GI tract—causes GI bleeding and is unique to SMAD4 cases. Personal or family history of colon polyps/cancer at young age. Overlap of two syndromes; distinguished from plain HHT by polyp findings (via endoscopy). Genetic test SMAD4.
HHT type 5—GDF2 (BMP9) mutation	GDF2 (AD)	615506	“HHT‐like”: may have epistaxis, telangiectasia, AVMs, but sometimes not all criteria are met. Essentially a mild HHT phenotype.	Milder or incomplete expression—e.g., patient has some nosebleeds and maybe one AVM but lacks widespread telangiectases or family history. Very rare (< 10 families known). Only distinguished by genetic testing GDF2. No unique clinical feature beyond a possibly lower penetrance of symptoms.
Blue rubber bleb nevus syndrome (BRBNS)	TEK (somatic)	112200	Mucocutaneous and GI bleeding from venous lesions	Multiple rubbery blue venous malformations, chronic anemia, no telangiectases, sporadic occurrence
PTEN hamartoma tumor syndrome (PHTS)	PTEN (AD)	168350/153480	Complex vascular anomalies	Macrocephaly, multiple hamartomas, mixed‐flow vascular lesions, high cancer risk, no mucocutaneous telangiectasia
Parkes Weber syndrome	RASA1 (AD)	608355	Multiple fast‐flow AVFs; limb overgrowth; cutaneous vascular stains sometimes misinterpreted as telangiectasia.	Limb hypertrophy; diffuse AVFs rather than discrete AVMs; capillary malformations with pale halos; minimal mucosal telangiectases; no pulmonary AVMs.
PHACES syndrome	Sporadic (developmental)	606519	Segmental facial capillary malformations can be mistaken for port‐wine or capillary telangiectasia	Posterior fossa anomalies, arterial dysplasia, cardiac defects, eye anomalies; usually unilateral large facial stain
Wyburn–Mason syndrome	Sporadic	180200	Facial vascular lesions + CNS AVMs	Ipsilateral retinal/midbrain AVMs; no mucosal telangiectasia; sporadic occurrence
Klippel–Trénaunay syndrome	Sporadic (PIK3CA‐related in some cases)	149000	Capillary malformations and limb overgrowth superficially mimic Parkes Weber	Venous malformations and lymphatic involvement predominate; no fast‐flow AVFs; no mucosal telangiectasia
Neurofibromatosis type 1	NF1 (AD)	162200	Vascular dysplasia (stenosis, aneurysms, rare AVMs)	Neurofibromas, café‐au‐lait spots, Lisch nodules, optic gliomas; no telangiectasia; vascular lesions less frequent
Cobb syndrome	Sporadic	NR	Cutaneous vascular lesions overlying spinal AVMs	Metameric distribution (skin lesion + spinal AVM); no visceral AVMs; no mucosal telangiectasia

**TABLE 2:** Cerebrovascular manifestations and imaging recommendations across HHT and HHT‐like syndromes.

Syndrome	Brain AVMs	Spinal AVMs	Pial AVMs	Dural AVFs	Vein of Galen malformation	Cerebral hemorrhage	Ischemic stroke	Cerebral aneurysms	Cavernomas/CCMs	Epilepsy	Headaches	Cognitive decline	Imaging recommendation	Developmental venous anomalies (DVA)	Flow type
HHT (ENG/ACVRL1)	Common (10%–20%)	Rare (1%)	Yes	Occasional	No	Yes (AVM rupture)	Paradoxical embolism from PAVM	Occasionally reported	Rarely associated	Post‐AVM rupture	Possible (esp. with AVMs)	Rare (secondary to AVMs)	Brain MRI/MRA in all suspected cases	No	High‐flow
CM‐AVM1 (RASA1)	Possible (30%)	Possible	Yes	Yes	Rare	Yes (AVM rupture)	Possible	Rare	Possible	AVM‐related	Yes (if AVMs present)	Rare	Brain/spine MRI, MRA for AVMs/fistulas	Possible	High‐flow
CM‐AVM2 (EPHB4)	Possible (15%)	Possible	Yes	Yes	Reported	Yes	Possible	Rare	Possible	AVM‐related	Yes (esp. if neonatal AVM)	Rare	Brain MRI (esp. if neonate/epistaxis/capillary malformations)	Possible	High‐flow
JP‐HHT (SMAD4)	As in HHT	As in HHT	Yes	Occasional	No	Yes	Possible	As in HHT	Possible	As in HHT	Yes	Possible	Standard HHT protocol imaging	Possible	High‐flow
HHT type 5 (GDF2)	Possible (low penetrance)	Rare/Unknown	Possible	Possible	Reported	Rare	Possible	Unknown	Possible	Possible	Possible (milder phenotype)	Unknown	MRI if incomplete HHT criteria or unexplained epistaxis	Unknown	Intermediate
BRBNS	Rare (venous malformations)	No	No	No	No	Rare (intracranial bleeds)	No	No	Rare	Rare	Possible	No	MRI if neurologic signs	No	Low‐flow
PTEN hamartoma tumor syndrome (PHTS)	Rare (PHOST lesions)	Rare	Rare	Rare	No	Rare (mixed‐flow lesions)	No	No	Occasional	Possible (lesion‐related)	No	Rare	MRI if neurologic symptoms	Frequent	Mixed‐flow
Parkes Weber syndrome (PWS)	Occasional	Rare	Sometimes	Frequent (limb > CNS)	No	Possible (AVM rupture)	Rare	Rare	No	If AVM present	Yes	Rare	MRI/MRA of brain and limbs if vascular lesion suspected	Possible	High‐flow
Cobb syndrome	No	Yes	No	Rare	No	No	No	No	No	No	No	No	MRI spine mandatory; brain MRI if neurologic symptoms	No	High‐flow
PHACES syndrome	Rare	No	No	No	No	Rare	Yes (arterial dysplasia)	Rare	No	Possible	Frequent	Possible	MRI/MRA brain in all cases	Occasional	Low‐flow
Klippel–Trénaunay syndrome	Rare	Rare	No	No	No	Rare	Rare	Rare	Rare	Rare	Occasional	Rare	MRI if neurological symptoms	Occasional	Low‐flow
Neurofibromatosis type 1	Rare	Rare	Rare	Rare	No	Rare	Possible (Moyamoya)	Rare	No	Rare	Rare	Rare	MIR/MRA if neurological symptoms or stroke	Rare	Low‐flow
Wyburn–Mason syndrome	Yes	No	Yes	No	No	Yes (AVM rupture)	Yes	Rare	No	Frequent	Common	Possible	MRI/MRA brain and orbits	Rare	High‐flow

## 3 Results

The results of this pictorial review are presented as a comparative synthesis of each syndrome's main features, with a specific focus on cerebrovascular manifestations and their distinction from classic HHT.

### 3.1 Wyburn–Mason Syndrome

Wyburn–Mason syndrome (WMS), also known as Bonnet‐Dechaume‐Blanc syndrome, is a rare, non‐hereditary neurocutaneous phakomatosis, typically recognized in childhood or adolescence [6, 7]. It is characterized by large congenital AVMs predominantly affecting the retina and ipsilateral brain. Patients typically exhibit unilateral retinal “racemose” angiomas alongside concurrent midbrain or cerebral AVMs [8, 9]. The disorder results from an embryonic error in vascular development and is classified as a cerebro‐facial arteriovenous metameric syndrome (CAMS2); no associated genetic variants have been identified. Clinically, patients with WMS may present with visual disturbances secondary to retinal AVM bleeding or with neurological deficits due to rupture of brain AVMs [8, 9]. In contrast to HHT, which is marked by multiple small mucocutaneous telangiectasias, capillary malformations, and recurrent epistaxis, WMS is defined by large, focal AVMs without mucosal involvement. Notably, WMS lacks epistaxis and pulmonary AVMs. Cerebrovascular complications are central to WMS, as midbrain‐localized cerebral AVMs can lead to stroke or hemorrhage [8, 9]. Thus, although both WMS and HHT involve brain AVMs, WMS is distinguished by its characteristic retinal involvement, non‐familial nature, and absence of diffuse telangiectasias [10].

### 3.2 Cobb Syndrome

Cobb syndrome (CS), also known as spinal arteriovenous metameric syndrome, is a rare, sporadic disorder characterized by the coexistence of cutaneous vascular nevi and spinal cord AVMs within the same metamere, usually presenting in infancy or childhood [11]. Patients typically present with a port‐wine–type stain or hemangiomatous lesion on the back, buttock, or flank, corresponding to an underlying intramedullary spinal AVM or arteriovenous fistula at the same dermatome level [12, 13]. The condition is thought to result from a defect in early embryonic angiogenesis that propagates along a metamere. Clinical presentations vary: some patients remain asymptomatic, while others develop spinal cord ischemia or hemorrhage, manifesting as back pain, radiculopathy, progressive weakness, or acute paraplegia. In some cases, patients may develop Foix‐Alajouanine syndrome, a subacute myelopathy caused by thrombosis of a spinal AVM [13]. In contrast to HHT, CS does not involve mucocutaneous telangiectasias or visceral AVMs, and there is no occurrence of epistaxis or pulmonary AVMs. The vascular anomalies in CS are strictly confined to the skin and spinal cord. Moreover, the genes implicated in HHT (*ENG*, *ACVRL1*) are not involved in CS. The key distinction lies in the anatomical distribution: HHT is characterized by multifocal telangiectasias and AVMs affecting the brain, lungs, and liver, whereas CS features segmental vascular lesions primarily restricted to the spinal cord [13, 14, 15]. Consequently, cerebrovascular complications in CS are limited to the spinal circulation, and cerebral AVMs or aneurysms are not expected, further differentiating CS from HHT.

### 3.3 Klippel–Trénaunay Syndrome

Klippel–Trénaunay syndrome (KTS) is a congenital overgrowth disorder classified within the *PIK3CA*‐related overgrowth spectrum (PROS), presenting at birth or early infancy [16]. It is defined by a triad consisting of capillary malformation (port‐wine stain), venous malformation, and soft‐tissue and/or bony hypertrophy of a limb. Lymphatic malformations may also be present [17, 18]. Most cases are caused by somatic activating mutations in the *PIK3CA* gene, leading to dysregulation of the PI3K‐AKT growth pathway. The extent of somatic mosaicism correlates with disease severity, supporting a topographic genotype–phenotype relationship. Unlike Parkes Weber syndrome (PWS) or HHT, the vascular anomalies in KTS are typically slow‐flow. Clinically, KTS usually presents at birth or in early infancy with a large hemangioma‐like port‐wine stain and an enlarged limb. It is sporadic and not inherited. In contrast to HHT, which features arteriovenous shunts and frequent epistaxis, KTS does not cause nosebleeds or pulmonary AVMs; instead, it predisposes to thrombotic complications due to its venous malformations [19, 20]. While CNS involvement is not a hallmark of KTS, CNS anomalies have been reported. Hemimegalencephaly is the most common brain finding, and rare cases have described intracranial vascular lesions [19, 20]. These include cavernous angiomas, cerebral or spinal AVMs, venous varices, and aneurysms. One case series documented spinal or cerebral AVMs and multiple aneurysms in KTS patients [19, 20]. Thus, although KTS primarily affects the limbs, its cerebrovascular manifestations are characterized by low‐flow malformations such as DVAs or cavernomas rather than AVMs that characterize HHT.

### 3.4 Neurofibromatosis Type 1

Neurofibromatosis type 1 (NF1) is an autosomal dominant neurocutaneous disorder caused by *NF1* gene variants encoding for neurofibromin, a Ras GTPase‐activating protein (OMIM: 162200). Although the genetic defect is present from birth, the clinical diagnosis is usually made in childhood, most often by 8–10 years of age. Most NF1 variants are loss‐of‐function, but some missense variants, including p.Arg1809, correlate with less severe phenotypes without plexiform neurofibromas [21]. Clinically, NF1 is characterized by café‐au‐lait macules, axillary freckling, cutaneous neurofibromas, Lisch nodules, and an increased risk of developing optic gliomas [6, 22]. Although NF1 is not primarily considered a vascular dysplasia syndrome, vascular involvement can occur due to neurofibromin deficiency in vascular cells. The cerebrovascular manifestations of NF1 are mainly occlusive and aneurysmal. Documented CNS vascular lesions include stenosis or occlusion of major cerebral arteries, often presenting with moya‐moya‐like patterns and collateral vessel formation, as well as fusiform or saccular aneurysms, and, more rarely, arteriovenous fistulas [23, 24]. Patients with NF1 have a higher risk of early‐onset moya‐moya syndrome or stroke compared to the general population. In contrast, the cerebrovascular profile of HHT is dominated by brain AVMs and associated hemorrhages. Unlike HHT, NF1 does not feature characteristic mucocutaneous telangiectasias or frequent epistaxis. Instead, vascular complications in NF1 more commonly lead to ischemic strokes or hemorrhages resulting from aneurysms [22, 23]. In summary, NF1 and HHT differ genetically (RAS/MAPK pathway vs. TGF‐β signaling) and pathologically. Routine screening for AVMs, as performed in HHT, is not indicated in NF1 patients; however, clinicians should remain vigilant for stroke risk due to moya‐moya‐like changes or aneurysms when managing individuals with NF1.

### 3.5 PHACE(S) Syndrome

PHACE(S) syndrome is a neurocutaneous syndrome of unknown etiology, characterized by a large segmental infantile hemangioma of the face/neck with anomalies involving posterior fossa, arteries, heart, eye, and sternal region [25, 26]. The condition is sporadic, presents in infancy, and shows a strong female predominance. The vascular abnormalities in PHACE are typically due to large‐artery dysplasia rather than telangiectatic shunts. Examples include agenesis or hypoplasia of the ipsilateral internal carotid artery, aberrant subclavian artery, aortic coarctation, and an increased risk of aneurysms [25, 26, 27]. Notably, cerebrovascular anomalies are present in up to 91% of PHACE patients. These often involve stenosis or absence of cerebral arteries on the side of the hemangioma, leading to a higher risk of ischemic stroke or arterial insufficiency [25, 26, 27]. Posterior fossa malformations, such as cerebellar hypoplasia and Dandy‐Walker malformation, are also frequent. In contrast to HHT, which features brain AVMs and telangiectasias, PHACE patients do not exhibit epistaxis or mucosal telangiectasias. Instead, they present with segmental hemangiomas and significant large‐vessel anomalies. The cerebrovascular features of PHACE are fundamentally different from the micro‐AVMs seen in HHT [25, 26, 27]. Recognition of a segmental facial or neck hemangioma in PHACE should prompt comprehensive angiographic evaluation of cerebral and cervical arteries, given the high prevalence of associated cerebrovascular abnormalities.

### 3.6 Capillary Malformation–Arteriovenous Malformation Syndromes (CM‐AVM)

*RASA1* (OMIM: 608354) and *EPHB4* (OMIM: 618196) gene defects cause autosomal‐dominant CM‐AVM syndromes, which combine clusters of cutaneous capillary malformations with fast‐flow arteriovenous shunts. *RASA1* loss‐of‐function is typically linked to PWS, characterized by capillary stains and limb‐based AV fistulas or overgrowth, while *EPHB4* variants are found in many RASA1‐negative cases [28, 29]. Both genes regulate endothelial signal transduction, and their disruption leads to defective capillary bed development and unregulated AVMs and AVFs. Although this process resembles the vascular lesions seen in HHT, it arises through distinct mechanisms [29, 30, 31]. Additionally, in humans, *RASA1* truncating variants are typically associated with extensive AVMs, while *EPHB4* missense variants cause CM‐AVM2, often with a milder, HHT‐like phenotype sparing pulmonary AVMs. Cutaneous capillary malformations are visible since birth or early childhood, while AVMs/AVFs can manifest later, often in childhood or adolescence. Clinically, patients often display multiple small skin lesions with pale halos and may develop larger AVMs in muscle, bone, viscera, or CNS. Intracranial and spinal AVFs frequently present in childhood, sometimes causing seizures, hemorrhage, or neurologic deficits. EPHB4 variants have been associated with Vein of Galen malformations, highlighting a spectrum ranging from superficial skin lesions to deep midline vascular anomalies [29, 30, 31]. Unlike HHT, recurrent nosebleeds and widespread mucosal telangiectasia are uncommon, though occasional minor mucosal involvement can occur. High‐output cardiac failure and bleeding may result from large shunts. Pulmonary and hepatic AVMs are rare, and paradoxical embolic stroke is infrequent. Management is individualized and includes embolization, surgery, or radiosurgery for symptomatic lesions, cosmetic treatment of skin malformations if desired, orthopedic care for limb overgrowth, and cardiology evaluation for cardiac failure [29, 30, 31]. Surveillance often involves brain and spinal MRI, even in asymptomatic individuals, and first‐degree relatives should be offered targeted assessment if a familial variant is known.

### 3.7 Parkes Weber Syndrome

Parkes Weber syndrome (OMIM: 608354) is a congenital disorder characterized by high‐flow vascular malformations, often apparent in infancy or early childhood. It typically presents with port‐wine stain capillary malformations and numerous micro‐arteriovenous fistulas (micro‐AVFs) within an affected limb, resulting in soft tissue and bone overgrowth [32, 33, 34]. Patients usually show symptoms at birth or in early childhood, including unilateral limb hypertrophy and pink‐red skin discoloration. Many cases of PWS are caused by mutations in the *RASA1* gene, which encodes the p120‐RasGAP protein. These variants disrupt normal vascular development and lead to high‐output arteriovenous shunting [35]. The arteriovenous shunts can be so extensive that they cause cardiac overload or bleeding complications. In contrast to HHT, which arises from defects in the TGF‐β signaling pathway and is marked by mucocutaneous telangiectasias and visceral AVMs, PWS involves fast‐flow vascular lesions primarily affecting the skin and limbs along with limb overgrowth. PWS patients do not typically experience recurrent nosebleeds or pulmonary AVMs, which are common features of HHT. Interestingly, some patients with RASA1 mutations have intracranial AVMs, including vein‐of‐Galen malformations, presenting in infancy [32, 35]. These high‐flow cerebral vascular malformations differ substantially from the slow‐flow telangiectasias characteristic of HHT. Although cerebrovascular involvement is rare in PWS, when present it usually consists of high‐flow brain AVMs, setting its vascular phenotype apart from that seen in HHT.

### 3.8 Polyposis/HHT Overlap Syndrome

The polyposis/HHT overlap syndrome (JP‐HHT) overlap syndrome is an autosomal dominant condition caused by heterozygous variants in the *SMAD4* gene. These variants lead to a combined clinical picture of juvenile polyposis syndrome (JPS) and features of HHT [36, 37]. Manifestations may appear in childhood, but HHT features (epistaxis, AVMs) often emerge in adolescence or young adulthood. Patients with *SMAD4* variants typically display signs of both disorders. Similar to classic HHT, they experience recurrent nosebleeds, mucocutaneous telangiectasias, and AVMs. In addition, they develop multiple juvenile polyps throughout the gastrointestinal tract [36]. These hamartomatous polyps frequently cause GI bleeding, abdominal pain, and anemia, and often present during childhood or adolescence with rectal bleeding. Polyps may be found in the stomach, small intestine, colon, and rectum. There is also an increased risk of gastrointestinal cancers due to the polyposis component. The clinical diagnostic criteria for JPS include having five or more juvenile polyps in the colon or rectum, juvenile polyps throughout the GI tract, or any juvenile polyp along with a family history of polyposis. When a patient meets the clinical criteria for HHT (e.g., recurrent epistaxis and mucocutaneous telangiectasias) and also has multiple juvenile polyps, the JP‐HHT overlap syndrome should be suspected. Genetic testing for *SMAD4* variants confirms the diagnosis [36, 37]. Current guidelines recommend testing for *SMAD4* in any individual presenting with HHT features plus GI polyps or a family history suggestive of polyposis. The cerebrovascular manifestations in JP‐HHT overlap syndrome are similar to those seen in HHT due to *ENG* or *ACVRL1* mutations, including pial AVMs, spinal AVMs, dural arteriovenous fistulas, and risks of both hemorrhagic and ischemic stroke [36]. Patients may also have cavernomas and aneurysms. Because of the high‐flow vascular lesions, imaging surveillance recommendations are the same as for classic HHT.

### 3.9 HHT Type 5 (BMP9/GDF2 Mutation)

Hereditary hemorrhagic telangiectasia type 5 is linked to mutations in the *GDF2* gene, which encodes bone morphogenetic protein 9 (BMP9). This form is inherited in an autosomal dominant pattern but is quite rare, accounting for less than 1% of all HHT cases. Despite being congenital, symptoms often do not appear until adolescence or early adulthood.

GDF2/BMP9 is a circulating ligand within the ALK1/ENG signaling pathway, and loss‐of‐function mutations result in an HHT‐like vascular phenotype [38, 39, 40]. Clinically, *GDF2* variants give rise to what is sometimes called HHT type 5, which resembles classical HHT but often appears milder or incomplete. Patients commonly present with recurrent epistaxis and may have mucocutaneous telangiectasias; however, many do not fulfill the full Curaçao diagnostic criteria for definite HHT [38, 39, 40]. For example, an individual might have frequent nosebleeds and a few telangiectases but lack visceral AVMs or a positive family history, thus falling into a “possible” rather than “definite” HHT category. In some cases, pulmonary hypertension has been reported, potentially related to high‐output cardiac states or hepatic AVMs [38, 39, 40]. Because of the often‐incomplete clinical presentation, diagnosis is typically confirmed via genetic testing. In individuals with HHT‐like features but negative results for *ENG*, *ACVRL1*, and *SMAD4* variants, *GDF2* sequencing may reveal a pathogenic variant, confirming the diagnosis of this HHT‐like telangiectasia syndrome. HHT type 5 is also associated with a cerebrovascular phenotype characterized by intermediate‐flow lesions [38, 39, 40]. Although less penetrant, affected individuals may develop pial AVMs, dural AVFs, and, in some cases, Vein of Galen malformations. DVAs and epilepsy have also been described but appear to be less common [38, 39, 40].

### 3.10 PTEN Hamartoma Tumor Syndrome

PTEN hamartoma tumor syndrome (PHTS), which includes Cowden syndrome (OMIM: 158350) and Bannayan‐Riley‐Ruvalcaba syndrome (OMIM: 153480), results from hyperactivation of the PI3K/AKT/mTOR signaling pathway. Patients typically present with macrocephaly, multiple hamartomas, vascular anomalies, and an increased risk of various cancers [41, 42, 43]. Generally, features arise in childhood, but tumor risk becomes most clinically relevant in adolescence or adulthood. A hallmark of PHTS is the presence of complex, mixed vascular malformations. These often appear as multifocal soft‐tissue masses containing venous channels, lymphatic components, and adipose tissue. The lesions frequently include fast‐flow channels and fibrofatty infiltration, making them “mixed‐flow” anomalies. Although often labeled as venous malformations on imaging, they frequently contain arteriovenous elements [41]. Beyond vascular issues, PHTS can also be associated with autism spectrum disorder, developmental delays, and gastrointestinal polyps. DVAs are common, while true AVMs are rare. However, patients may develop PTEN hamartomas of soft tissue (PHOST), which can involve muscle, subcutaneous tissue, and even extend into the mediastinum or head and neck regions [41, 42, 43]. Routine neurologic imaging is not recommended in asymptomatic individuals. Instead, surveillance focuses on cancer risk reduction: for example, annual thyroid ultrasounds, breast MRI starting at age 30, renal ultrasounds from age 40, and colonoscopy tailored to polyp findings. Management of vascular lesions is individualized. Given that *PTEN* mutations lead to PI3K/AKT/mTOR pathway hyperactivation, mTOR inhibitors have been used off‐label with some success in treating vascular tumors and malformations [41, 42, 43].

### 3.11 Blue Rubber Bleb Nevus Syndrome

Rubber bleb nevus syndrome (BRBNS, OMIM: 112200) is a sporadic venous malformation disorder caused by somatic activating mutations in the *TEK* (*TIE2*) gene, often presenting congenitally or early in childhood [44, 45, 46]. It is characterized by the presence of multiple, rubbery, blue‐colored venous malformations, often referred to as “blebs,” that primarily involve the skin and gastrointestinal tract. These lesions are low‐flow venous malformations, though they can occasionally involve other organs such as the lungs, muscles, and, less frequently, the CNS [44, 47, 48]. While intracranial venous malformations are rare, they have been reported. Diagnosis is typically based on recognizing the distinctive cutaneous lesions during physical examination, combined with endoscopy or MRI to identify internal vascular malformations. Genetic testing of tissue from the lesions may be performed to confirm *TEK* variants when necessary [44, 47, 48]. Management focuses on treating complications such as chronic anemia, which may require iron supplementation or blood transfusions. Lesions that are accessible can be addressed with sclerotherapy or surgical excision, while gastrointestinal blebs may be managed by endoscopic removal or coagulation. Care is ideally provided through a multidisciplinary team approach.

## 4 Discussion

HHT exemplifies a high‐flow vascular dysplasia with an elevated hemorrhagic tendency, setting it apart from many mimicking conditions. In classic HHT, fragile mucosal telangiectasias and visceral AVMs lead to recurrent bleeding and can cause life‐threatening intracranial hemorrhage [49]. Similar high‐flow shunts are seen in CM‐AVM syndromes and PWS, where large or multiple cerebral AVMs and AVFs increase the risk of hemorrhagic stroke, sometimes even in infancy [30, 35]. WMS represents another prototypical high‐flow mimic: these sporadic, non‐hereditary CAMS2 lesions often produce large pial AVMs and retinal “racemose” angiomas, typically presenting with epilepsy or stroke [9]. In all these disorders, the cerebrovascular phenotype is dominated by fast‐flow arteriovenous shunts and hemorrhage risk. JP‐HHT shares this AVM risk profile but also introduces a gastrointestinal polyp burden [37]. By contrast, some HHT‐like conditions predominantly predispose to ischemic events rather than hemorrhage. NF1 is associated with occlusive arteriopathy, often manifesting as moya‐moya pattern stenoses or fusiform aneurysms, which elevate ischemic stroke risk [23]. NF1 patients face a significantly increased likelihood of both intracerebral hemorrhage in childhood and ischemic stroke compared to the general population [23, 24]. PHACE(S) syndrome also involves large‐artery dysplasia, typically adjacent to segmental facial hemangiomas [25, 27]. Up to 83%–91% of PHACE patients harbor cerebrovascular anomalies predisposing them to focal hypoperfusion, with strokes more often ischemic due to arterial insufficiency rather than hemorrhage [25, 27]. Consequently, in both NF1 and PHACE, clinicians prioritize arterial imaging and stroke prevention over AVM screening. Other mimics primarily cause venous or low‐flow malformations, posing a lower risk to the CNS. KTS, a PIK3CA‐related overgrowth disorder, involves capillary stains, venous malformations, and limb hypertrophy [18, 20]. Lesions are typically slow‐flow, leading to venous hypertension and thrombosis rather than hemorrhage. KTS patients often experience deep venous thromboses, pulmonary emboli, and gastrointestinal bleeding from venous anomalies, while large brain AVMs are rare. BRBNS also causes multifocal venous malformations (“blebs”) affecting the skin and viscera [18, 20]; CNS involvement is rare, and morbidity is mainly driven by chronic GI bleeding [47, 48]. Cowden syndrome and related PHTS cause complex mixed venolymphatic malformations, macrocephaly, and tumors [43]. These typically appear as venous‐channel hamartomas or fibrofatty vascular masses rather than discrete AVMs. True CNS AVMs are uncommon, so routine brain MRI is not recommended unless symptoms arise, and management focuses on oncologic surveillance [41, 42, 43]. Finally, CS is a segmental, non‐hereditary disorder marked by skin capillary/venous nevi overlying a same‐level spinal AVM. It leads to spinal cord ischemia or hemorrhage but does not involve the brain [14, 15].

### 4.1 Epidemiology

These syndromes vary greatly in prevalence HHT affects approximately 1 in 5000–8000 individuals worldwide and occurs across all ethnicities [1, 5]. NF1 is similarly common, affecting about 1 in 3000–4000 individuals [22]. CM‐AVM syndromes are rarer, estimated at 1 in 20,000 to 1 in 12,000, reflecting under‐recognition of vascular birthmarks [30]. KTS is rare (2–5 per 100,000), with no sex preference [18] and Cowden/PHTS affects roughly 1 in 200,000 [42]. The remaining syndromes are exceptionally rare: PHACE syndrome has an incidence below 1 per million (with about 300 reported cases, 90% in females). BRBNS has around 200 reported cases [47]; WMS fewer than 100 [9]; Cobb syndrome under 50 [15]. GDF2‐related HHT is extremely rare (under 1% of HHT cases) [39], and JP‐HHT comprises about 1% of HHT probands [37]. All these conditions are reported worldwide with no strong racial predilection, though female predominance is notable in PHACE and mosaic overgrowth syndromes.

### 4.2 Diagnostic Considerations

Accurate differentiation relies on integrating clinical, imaging, and genetic data. HHT is diagnosed using Curaçao criteria and confirmed through *ENG* or *ACVRL1* testing; *SMAD4* sequencing is pursued in cases with overlapping juvenile polyposis. GDF2 variants cause a milder telangiectasia syndrome (*HHT* type 5), and negative results for classic HHT genes in a suspect patient should prompt GDF2 testing [39, 40]. In CM‐AVM, characteristic multiple pale capillary malformations with pulsatile elements suggest RASA1 or EPHB4 variants [28]. PWS falls under the *RASA1* spectrum, with genetic overlap [33]. KTS is usually sporadic and involves somatic *PIK3CA* variants; mosaicism complicates genetic testing, often requiring lesion biopsy for confirmation [18]. NF1 is diagnosed by clinical criteria and typically confirmed by *NF1* gene sequencing [22]. PHACE(S) lacks a known genetic marker and is defined by segmental facial hemangiomas plus major brain or vascular anomalies; diagnosis relies heavily on dermatologic clues. Once suspected, vascular imaging is crucial, as up to 90% of patients have intracranial arterial dysplasia [27]. Additionally, although all these syndromes are congenital, their clinical onset varies. KTS, PWS, CS, PHACES, CM‐AVM, and BRBNS usually present in infancy or early childhood with cutaneous or vascular anomalies. NF1 [22] and PHTS [41], while also congenital, often manifest progressively, peaking in late childhood. HHT [49], JP‐HHT [37], and HHT5 [39, 40] may remain clinically silent until adolescence or early adulthood, when recurrent epistaxis or visceral AVMs appear. WMS can also stay asymptomatic until later in life [9].

So far, clear genotype–phenotype correlations have been demonstrated mainly in HHT and CM‐AVM. In HHT, *ENG* variants are typically linked with pulmonary and cerebral AVMs, while *ACVRL1* variants more often lead to hepatic involvement; truncating changes also tend to produce more severe disease than missense ones [36]. In CM‐AVM, *RASA1* loss‐of‐function variants predispose to multifocal lesions including intracranial AVMs, whereas *EPHB4* missense variants are associated with milder presentations that can resemble HHT but lack pulmonary AVMs [28, 29]. For the other conditions the data are more limited and correlations less robust, while in PHACES, WMS, and CS no causative gene has been identified to date.

### 4.3 Imaging Strategies

Given the high AVM risk in HHT and other fast‐flow syndromes, routine brain and spinal MRI/MRA is recommended for patients and at‐risk relatives. In CM‐AVM and PWS, early neurologic imaging is similarly essential since cerebrospinal AVFs and Vein of Galen malformations can appear in infancy [28, 29]. By contrast, NF1 patients do not undergo routine screening unless they develop stroke symptoms or new neurologic deficits; imaging often reveals stenoses or aneurysms [22]. KTS evaluation focuses on limb vasculature and orthopedic assessment rather than brain imaging unless unexplained symptoms appear [18]. BRBNS typically requires endoscopic or CT angiographic surveys of the gastrointestinal tract, with brain MRI reserved for neurological complaints [46]. Across all syndromes, interdisciplinary genetic panels and careful radiologic evaluation are critical to avoid missed or overlapping diagnoses.

### 4.4 Outcomes and Prognosis

Neurologic outcomes vary widely. HHT patients face a risk of hemorrhagic stroke from AVMs and paradoxical embolic strokes from pulmonary AVMs. Early AVM detection and embolization significantly improve prognosis; with appropriate management, life expectancy is only modestly reduced [1, 5]. NF1 patients, however, have a reduced lifespan by about 10–15 years, largely due to tumors and vascular complications. The high stroke risk in NF1, particularly in children, highlights the need for vigilance [24]. PHACE can be life‐threatening in infancy if major vascular anomalies are not identified but survivors often do well with appropriate vascular intervention and hemangioma treatment. Careful aspirin use and neurosurgical follow‐up can help prevent strokes [27]. For slow‐flow syndromes like KTS and BRBNS, CNS outcomes are generally favorable. KTS patients rarely experience CNS bleeds; morbidity usually results from lymphedema, thrombosis, and orthopedic complications [16, 45, 47]. BRBNS patients have normal life expectancy but can suffer severe anemia from chronic GI bleeding. CNS involvement is rare and outcomes depend on lesion characteristics. PHTS patients are more challenged by oncologic than neurologic issues [41]. Multifocal vascular lesions rarely cause strokes, but lifelong cancer surveillance is critical. Among mosaic overgrowth syndromes, prognosis varies based on the severity of segmental vascular lesions; spinal forms like Cobb syndrome can cause paralysis or myelopathy [15]. Overall, most “HHT‐like” disorders lack the universal hemorrhagic threat of HHT, but each carries its own specific risks: JP‐HHT adds gastrointestinal cancer risk [36]; KTS predisposes to thrombosis and possible limb loss [17]; PHACE can lead to stroke and vision loss; and PHTS increases breast and thyroid cancer risks [25]. Besides standard management approaches, recent pharmacologic advances have emerged: two Cobb syndrome patients harboring KRAS mutations responded remarkably well to MEK inhibitor therapy with trametinib. This study by Sun et al. [50] has paved the way for syndrome‐specific targeted therapies, highlighting the potential of precision medicine in this context.

### 4.5 Clinical and Radiological Versus Genetic Testing

Clinical evaluation and radiological assessment remain the first steps in diagnosing HHT and related vascular syndromes, as they allow rapid recognition of characteristic phenotypes and urgent management of cerebrovascular risk. However, these methods have obvious drawbacks: imaging results frequently overlap between disorders, which can cause diagnostic uncertainty, and clinical features may be subtle or lacking. Because genetic testing can identify the underlying molecular defect and help differentiate HHT from phenotypically similar conditions, it offers a significant advantage in these situations. With sequencing increasingly available in urgent clinical settings, molecular confirmation can now be obtained more rapidly, supporting early diagnosis, risk stratification, and surveillance of at‐risk relatives. For these reasons, the integration of clinical, imaging, and genetic data offers the most reliable strategy to achieve diagnostic accuracy and guide personalized care.

## 5 Limitations

This review has several limitations inherent to its narrative design. It is not a systematic review or meta‐analysis, and source selection was based on subjective relevance rather than formal quality assessment. The scarcity of large cohort studies means prevalence and outcome data often derive from small series or single‐center experiences, limiting generalizability. Additionally, inconsistent imaging protocols and diagnostic criteria across syndromes introduce ascertainment bias when comparing cerebrovascular risks. Finally, this review emphasizes clinical and radiologic perspectives rather than quantitative analysis of genetic variant pathogenicity or penetrance. Future prospective registries and standardized imaging studies will be vital to confirm and expand these observations.

## 6 Conclusion

Although several syndromes can mimic aspects of HHT, most do not share its characteristic high‐flow arteriovenous shunts and hemorrhagic complications. Careful differentiation through genetic testing and targeted neuroimaging is essential for accurate diagnosis and personalized management.

## Author Contributions

All authors involved in conception and design, data collection, data analysis, drafting, draft revision, and approval of final version.

## Funding

The authors have nothing to report.

## Ethics Statement

IRB approval was not required for systematic review.

## Consent

The authors have nothing to report.

## Conflicts of Interest

The authors declare no conflicts of interest.

## Data Availability

The authors have nothing to report.
